# Red Blood Cell DNA Capture and Delivery Drives Host Responses During Polymicrobial Sepsis

**DOI:** 10.21203/rs.3.rs-2592196/v2

**Published:** 2024-04-23

**Authors:** Long Kwan Lam, Nathan Klingensmith, Layal Sayegh, Emily Oatman, Joshua Jose, Christopher Cosgriff, Kaitlyn Eckart, John McGinnis, Piyush Ranjan, Matthew Lanza, Nadir Yehya, Nuala Meyer, Robert Dickson, Nilam Mangalmurti

## Abstract

Red blood cells (RBCs), traditionally recognized for their role in transporting oxygen, play a pivotal role in the body's immune response by expressing TLR9 and scavenging excess host cell-free DNA. DNA capture by RBCs leads to accelerated RBC clearance and triggers inflammation. Whether RBCs can also acquire microbial DNA during infections is unknown. Murine RBCs acquire microbial DNA in vitro and bacterial-DNA-induced macrophage activation was augmented by WT but not TLR9-deleted RBCs. In a mouse model of polymicrobial sepsis, RBC-bound bacterial DNA was elevated in WT but not in erythroid TLR9-deleted mice. Plasma cytokine analysis revealed distinct sepsis endotypes, characterized by persistent hypothermia and hyperinflammation in the most severely affected subjects. RBC-TLR9 deletion attenuated plasma and tissue IL-6 production in the most severe endotype. Parallel findings in human subjects confirmed that RBCs from septic patients harbored more bacterial DNA compared to healthy individuals. Further analysis through 16S sequencing of RBC-bound DNA illustrated distinct microbial communities, with RBC-bound DNA composition correlating with plasma IL-6 in patients with sepsis. Collectively, these findings unveil RBCs as overlooked reservoirs and couriers of microbial DNA, capable of influencing host inflammatory responses in sepsis.

### Introduction

Recognition of microbial-derived nucleic acid by cytosolic and endosomal receptors is vital for initiating inflammatory responses essential for host defense. We have recently discovered that mammalian red blood cells (RBCs) express the nucleic acid-sensing receptor TLR9 and can bind mitochondrial DNA, functioning as immune sentinels by triggering rapid senescence and loss of self, with consequent clearance and innate immune activation upon excess DNA binding.(1) Collectively, these findings suggest that circulating RBCs modulate host immune responses during inflammatory states, yet whether RBCs acquire bacterial DNA during sepsis remains unknown.

While elevated plasma cell-free CpG-containing mitochondrial DNA is a hallmark of critical illness syndromes, including sepsis and trauma, an emerging body of literature has demonstrated elevated bacterial DNA in the circulation during sepsis and pneumonia.(2, 3) In light of the accumulating evidence demonstrating the association of circulating microbial DNA and host inflammatory responses and the plausible interaction between RBCs and bacteria in the vascular compartment, we asked whether RBCs could acquire microbial DNA and orchestrate distinct host inflammatory responses.(4) In a murine model of polymicrobial sepsis we show that RBCs capture microbial DNA during sepsis and that RBC-TLR9-mediated DNA delivery drives hyperinflammation. Moreover, RBCs from critically ill patients with sepsis harbor distinct microbial DNA composition compared to those from healthy donors, which correlate with the systemic inflammatory response. Thus, the process of RBC-mediated DNA capture and delivery shapes diverse host inflammatory responses during sepsis.

### Results

*CS injection recapitulates human sepsis features and RBC-bound bacterial DNA is elevated following CS-induced sepsis*

Although no pre-clinical model can recapitulate all of the features of a clinical syndrome, the cecal slurry (CS) model of polymicrobial sepsis captures the hallmark features of sepsis, including organ dysfunction, temperature disruption, and cytokine storm.(5, 6) To investigate the role of RBC-DNA capture in sepsis, we induced CS sepsis in WT mice and mice in which TLR9 was deleted in erythrocytes ($Ery^{tlr9-/-}$ mice), Suppl Fig 1.(1) CS injection induced weight loss and hypothermia in all mice (Fig. 1a-c). At early time points but not later time points, $Ery^{tlr9-/-}$ mice demonstrated increased hypothermia when compared with WT mice (Fig. 1b). Although a subset of WT mice never developed hypothermia, all $Ery^{tlr9-/-}$ mice developed hypothermia (Fig. 1c, P=0.035). CS injection led to mortality in WT and $Ery^{tlr9-/-}$ mice, (Fig 1d). Bacterial dissemination was measured by quantifying CFUs in distant organs following CS injection. We observed increased spleen bacterial dissemination in $Ery^{tlr9-/-}$ mice compared to WT mice, P=0.03, Table 1.

We next examined the organs of mice subjected to the sepsis model 24 hours following CS injection. Consistent with previous reports, splenic injury was characterized by neutrophilic inflammation, lymphocyte death, and germinal hyperplasia (Supplemental Fig. 1a&b, Supplemental Table 1). Liver injury, characterized by hepatocyte apoptosis, oval cell hyperplasia, and focal necrosis, was also present (Supplemental Fig. 1c&d, Supplemental Table 1). Notably, liver microabcesses were present in the two of the $Ery^{tlr9-/-}$ mice but none of the WT mice.

We next asked if RBCs bound live bacteria during the CS model, RBCs were not associated with live bacteria as we could not culture bacteria from purified RBCs obtained from control or CS-injected mice (Supplemental Fig. 2e). Because we had previously observed RBC acquisition of mtDNA during human and murine sepsis(1), we asked whether RBCs acquired bacterial DNA in a murine model of polymicrobial sepsis by performing qPCR for 16S on RBCs obtained from mice subjected to the cecal slurry model of sepsis. Consistent with our observation of increased mtDNA acquisition during sepsis, 16S was elevated on RBCs in WT mice six hours following the induction of CS-induced sepsis. RBCs from the $Ery^{tlr9-/-}$ mice did not acquire microbial DNA after CS injection, demonstrating that RBCs-acquire microbial DNA during sepsis via TLR9 (Fig. 1e).

Since we previously observed morphologic changes in human RBCs following excess DNA binding(1) and we detected elevated microbial DNA bound to RBCs during sepsis, we asked if RBC morphology is altered following the induction of CS-induced sepsis. Notably, $Ery^{tlr9-/-}$ RBCs remained morphologically intact, whereas echinocytes and loss of biconcave shape were observed in WT RBCs (Fig. 1f and g). These findings suggest that RBC morphological changes observed during sepsis depend on RBC-TLR9.

*Host Cytokine Responses are Heterogenous and Dependent on RBC-TLR9*

We next measured plasma cytokines following CS injection. We observed substantial heterogeneity in plasma cytokine production in the CS-injected mice (Fig 2a). To better dissect the heterogeneity observed, we applied uniform manifold approximation and projection (UMAP) in order to visualize the nine measured cytokines in a two-dimensional space. Three distinct clusters emerged in this projection, which discriminated between persistent temperature phenotype and controls (Fig 2b). Cluster 1 was defined by persistent hypothermia and associated with increased organ dissemination, cytokines, and organ injury (Fig 2b, Supplemental Fig. 2). Cluster 2, was defined by transient hypothermia and was associated with decreased organ dissemination, decreased organ injury, and decreased cytokine production (Fig 2b, Supplemental Fig. 2). To visualize the relative concentration of the cytokines in each cluster, we fit a kernel density estimator to the UMAP space weighted by each of the log-normalized features revealing Cluster 1 to be enriched with the proinflammatory cytokines (Fig 2c). Agglomerative clustering on the nine cytokines recovered the same clusters (Fig 2d) and exemplified that there were two distinct cytokine expression patterns in response to the same insult, and these patterns correlated well with the temperature response phenotype.

Having identified this temperature response endotype, we re-classified mice on this clinically identifiable trait and compared the cytokine response by strain within each endotype. Analysis based on the hypothermic cluster revealed aberrant cytokine production in the persistently hypothermic $Ery^{tlr9-/-}$ mice when compared with persistently hypothermic WT mice. We observed attenuated plasma IL-6, IL-10, and IL-1β in the $Ery^{tlr9-/-}$ mice compared to WT mice (Fig. 2e). Spleen and liver cytokine transcripts were examined by qPCR. In the spleen we observed attenuated IL-6 and IL-10, while in the liver we observed attenuated IL-6, IL-1β and IL-10 production (Fig. 2f-i). Spleen IL-12 family cytokines transcripts were also attenuated in the $Ery^{tlr9-/-}$ mice (Supplemental Fig. 2c). Because the erythropoietin receptor is present on some non-erythroid populations including red pulp macrophages (RPM) and we observed striking differences in dissemination and cytokine production in the spleens between WT and $Ery^{tlr9-/-}$

mice, we analyzed red pulp macrophages from WT and Ery$^{tlr9-/-}$ mice to ascertain whether TLR9 was deleted in RPM.(7) As seen in Supplemental Fig. 3, RPMs in Ery$^{tlr9-/-}$ mice did not demonstrate a reduction in TLR9. Thus, the differences in dissemination and tissue cytokine production appear to be a result of RBC-TLR9 deletion.

We examined correlations between tissue and plasma cytokines to determine if plasma cytokines reflect tissue cytokine production. Plasma IL-6, TNFα, and IL-10 correlated with spleen and liver cytokine generation (Supplemental Table 2). However, spleen and liver IL-1β were concordant with plasma levels in WT but not Ery$^{tlr9-/-}$ mice, and tissue IFNγ was discordant with plasma in both WT and erythroid TLR9 deficient mice (Supplemental Table 2), suggesting that plasma IFNγ may not be a surrogate for tissue-level pathology during sepsis. Because we observed differences in organ dissemination between WT and Ery$^{tlr9-/-}$ mice, we next asked whether tissue cytokines correlated with bacterial dissemination in remote organs in WT and Ery$^{tlr9 -/-}$ mice. We observed significant correlations between tissue dissemination and cytokines in the spleen and liver of WT and Ery$^{tlr9-/-}$ mice. However, the cytokine and bacterial dissemination correlations differed between the two groups (Fig. 2g and i) with signficant associations between tissue IL-6 and IL-1β cytokine production and dissemination observed in WT but not Ery$^{tlr9-/-}$ mice. Collectively, these findings suggest that RBC-TLR9-mediated DNA delivery may account for heterogeneous host responses to identical stimuli.

*RBCs deliver DNA to immune cells, initiating inflammatory responses*

To validate our observations in the sepsis model, we asked whether RBCs could increase the delivery of microbial DNA to immune cells. We first assessed the ability of RBCs to sequester pathogen-derived DNA, RBCs were incubated with known quantities of bacterial DNA and then assayed for bacterial DNA acquisition by qPCR. *In vitro*, murine RBCs bound genomic DNA from common bacterial pathogens (Supplemental Fig. 4a). We next treated peritoneal macrophages with genomic DNA from Staphylococcus aureus (Sa), Pseudomonas aeruginosa (PsA), or the immunostimulatory ODN, CpG 1826 or DNA-treated WT or TLR9 deficient RBCs. Microbial DNA alone did not result in macrophage activation as measured by TNFα secretion. While microbial DNA alone did not lead to TNFα release, Sa DNA carrying WT RBCs but not Sa-DNA treated TLR9 deficient RBCs induced robust TNFα secretion (Fig. 3a), likewise PsA DNA alone did not result in macrophage activation and TNFα release. However, immunostimulatory CpG did result in robust macrophage activation. WT RBCs but not $Ery^{tlr9-/-}$ RBCs augmented CpG-mediated macrophage activation. To verify that our findings were not due to the inflammatory effects of heme, we measured cell-free hemoglobin in the supernatant of RBC-treated macrophages. We did not observe increased hemolysis in the microbial DNA-treated RBC group, Supplemental Fig. 4b.

We previously found that CpG-treated RBCs can trigger innate inflammatory responses in naïve mice.(1) We now asked whether RBC TLR9 mediated DNA delivery to remote organs in naïve mice. WT RBCs and TLR9 KO RBCs alone did not lead to liver inflammation as measured by increased neutrophil recruitment, while CpG did lead to increased liver neutrophil recruitment. CpG-treated WT RBCs increased liver neutrophils when compared with RBCs or CpG alone, while CpG-treated TLR9 KO RBCs did not increase liver neutrophil recruitment (Fig. 3b and c). These data would suggest that RBCs through TLR9 can deliver immunostimulatory DNA to remote organs, triggering inflammatory cell recruitment. Consistent with these results, infusion of CpG-treated TLR9 KO RBCs resulted in decreased plasma IL-6 production when compared with infusion of CpG-treated WT RBCs. (Fig. 3d). Collectively, these findings suggested that RBCs are capable of supplying microbial DNA to immune cells and inciting inflammatory cytokine production.

*RBC-bound microbial DNA community richness but not DNA burden correlates with IL-6 in human sepsis*

To determine whether our pre-clinical observations of RBC-mediated immunoregulation through DNA-binding and delivery are germane to human sepsis, we asked whether human RBCs are associated with microbial DNA in health and in sepsis (Fig. 4). First, we incubated human RBCs with *Legionella* sp. DNA *in vitro* to confirm that human RBCs are capable of binding bacterial DNA. When we assayed these RBCs using 16S rRNA gene amplicon sequencing, 97.5% (SD 1.5%) of all RBC-associated sequences were classified as *Legionella* sp. (Fig. 4a). We confirmed microbial DNA binding to RBCs with qPCR following incubation of genomic DNA from different pathogenic bacterial strains with human RBCs (Supplemental Fig. 5). We thus concluded that human RBCs are capable of binding bacterial DNA and being assayed via 16S rRNA gene amplicon sequence.

We next investigated the *quantity* and *diversity* of bacterial DNA associated with human RBCs using qPCR of the bacterial 16S gene. As shown in Figure 4b, human RBCs contained more detectable bacterial DNA than our negative controls (buffer controls, $P<0.0001$, sterile water, $P<0.0001$). When we compared the quantity of bacterial DNA detected on RBCs from patients with and without sepsis, we found a greater quantity of bacterial DNA on RBCs from patients with sepsis (P = 0.0052). Baseline characteristics of the sepsis cohort are found in Table 2. Next, using 16S rRNA gene amplicon sequencing, we compared the *community diversity* of bacterial DNA associated with human RBCs. As shown in Figure 4c, community diversity (both as measured using the Shannon Diversity Index and by community richness) was greater in human RBC-associated bacterial DNA than in negative control specimens. Neither diversity index differed across humans with and without sepsis. We thus concluded that human RBCs contain more bacterial DNA and greater bacterial diversity than do background control specimens, and the quantity of RBC-associated bacterial DNA is greater in sepsis than in health.

We then assessed the *community composition* of bacterial DNA detected in association with human RBCs (Fig. 4d-f). Whether visualized via principal components analysis (Figure 4d) or rank abundance (Figure 4e) or tested using permutational multivariate ANOVA (PERMANOVA), we found distinct bacterial communities when comparing negative controls and human RBCs, both in health and sepsis (P<0.0001 for both, PERMANOVA). Communities detected in RBCs from healthy subjects differed from those detected in RBCs from subjects with sepsis (P=0.007, PERMANOVA). As shown via rank abundance analysis (Figure 4e), some contaminant taxa detected in negative controls (e.g., *Comamonadaceae*) were detected in RBCs from healthy patients and patients with sepsis, though at a lower relative abundance (>50% in negative controls vs 10-15% in RBC specimens). In contrast, numerous taxa were detected in RBC-associated communities that were minimally present in negative controls (e.g., *Pseudomonadaceae*, *Mycobacteriaceae*). We thus concluded that while, as a low-biomass specimen, bacterial DNA associated with human RBCs is vulnerable to sequencing contamination, it also has a distinct bacterial signature that cannot be wholly explained via contamination.

We next determined the source of this distinct bacterial signal in RBC-associated bacterial DNA (Figure 4f). We directly compared prominent bacterial families detected in RBCs, as compared to negative control specimens, grouped by probable source community. Much of the bacterial signal was suspicious for procedural or sequencing contamination, evidenced by the high relative abundance of *Comamonadaceae* spp. (detected in negative controls) and *Flavobacteriaceae* spp. (minimally present in negative controls but taxonomically suggestive of an occult source of contamination). However, we found some bacterial taxa enriched in RBC-associated DNA suggestive of probable gut origin (e.g., *Lachnospiraceae*, *Clostridiaceae*, and *Enterococcaceae* spp.). Finally, some bacterial taxa were classified as potential pathogens (e.g., *Pseudomonadaceae* spp., *Staphylococcaceae* spp., *Streptococcacaeae* spp., *Actinomycetaceae* spp., and *Mycobacteriaceae* spp.). Of these, *Streptococcacaeae* spp., *Actinomycetaceae* spp., and *Mycobacteriaceae* spp. were most enriched in RBCs from sepsis patients relative to those from healthy subjects. Importantly, among sepsis patients, neither the diversity, quantity, or community composition of RBC-associated bacterial DNA differed across patients with and without culture-identified bacteremia (Supplemental Fig. 5, $P>0.05$ for all comparisons).

We next compared RBC-associated microbiota with patients' plasma concentrations of IL-6. We found that the community richness of RBC-associated bacterial DNA (the number of unique bacterial taxa detected) was positively correlated with patients' IL-6 concentrations, explaining 12% of patient variation in this inflammatory cytokine (Fig. 4g). Using PERMANOVA, we found that the community composition of RBC-associated bacteria was correlated with patients' IL-6 concentrations, both at the OTU and family level of taxonomic classification (P = 0.03 and 0.01, respectively).

Taken together, we concluded that human RBCs are capable of binding bacterial DNA and contain a greater quantity and diversity of bacterial DNA than do negative control specimens. The identity of RBC-associated bacterial DNA is influenced – but not entirely explained by – procedural and sequencing contamination, and the identity of bacterial DNA in RBCs from patients with sepsis differs from that of healthy subjects and is correlated with systemic IL-6 concentrations.

**Discussion**

In this study, we identify RBCs as critical regulators of the host inflammatory response during sepsis. Using a pre-clinical model of sepsis and genetic deletion of erythrocyte TLR9, we demonstrate that circulating red cell-mediated DNA delivery drives heterogeneous host inflammatory responses through DNA capture and delivery to remote organs. *In vitro*, RBCs bind microbial DNA and increase DNA delivery to phagocytes triggering inflammation. Moreover, RBCs from patients with sepsis reveal distinct microbial DNA profiles and we identify red blood cells as a distinct reservoir for microbial DNA. These data demonstrate a novel role for circulating RBCs as couriers of microbial DNA, capable of inciting heterogeneous host inflammatory responses during sepsis.

There is renewed interest in anti-inflammatory treatments for sepsis following the recent success of anti-inflammatory treatments for COVID-associated sepsis and ARDS.(8-10) Clinical trials of anti-IL-6 and re-analysis of IL-1RA for sepsis have yielded insight into the importance of these pathways in the host inflammatory response to pathogens in specific subsets of patients.(11) However, large trials of anti-inflammatories for sepsis have yet to show a mortality benefit. Discussions surrounding the failure to translate pre-clinical trials to successful therapies for sepsis have centered around the heterogeneous nature of this clinical syndrome and the inability of animal models to fully demonstrate the clinical features of this complex syndrome. Thus, animal modeling of clinical syndromes such as sepsis has been intensely scrutinized.(12) Identifying novel mechanisms that trigger inflammation within sepsis patients offers the promise of precision-medicine-based approaches for this complex syndrome. Here, using a pre-clinical model of sepsis, erythroid TLR9 deficient mice, *in vitro* studies, and human subjects, we demonstrate that animal models can recapitulate key clinical features of sepsis and even provide insight into heterogeneous host responses. There was substantial heterogeneity in the inflammatory response to CS injection in wild-type and in $Ery^{tlr9-/-}$ knockout mice. Moreover, distinct differences in the tissue and systemic inflammatory cytokine production were observed in the absence of RBC-TLR9 in the persistently hypothermic cluster. This observation implies that the mechanism of red blood cell-mediated DNA delivery plays a significant role in eliciting part of the host's inflammatory response in this severe endotype. These observations were only possible after our unbiased analysis of plasma cytokines demonstrated differential clustering based on the persistent hypothermic response leading to a re-analysis of subjects in this temperature endotype. These findings are consistent with recent reports suggesting temperature trajectories predict clinical outcomes.(4, 13) Together, these results underscore the critical role of pre-clinical models in identifying mechanistic elements (ie RBC-TLR9) that may drive differential host inflammatory responses and identify RBC-TLR9 mediated DNA regulation as a potentially treatable trait in sepsis.

In the polymicrobial sepsis model, we observed a significant reduction in cytokine production in the plasma and spleen, alongside increased organ dissemination in mice that lacked erythrocyte TLR9. In vitro experiments showed that wild-type (WT) red blood cells (RBCs) could activate macrophages in the presence of bacterial DNA, whereas RBCs from erythrocyte-specific TLR9 knockout (Ery tlr9) mice did not. These data indicate that TLR9-mediated DNA delivery by RBCs plays a crucial role in regulating the host's inflammatory response. Additionally, in a focused model examining DNA delivery by RBCs, the absence of RBC TLR9 impaired neutrophil recruitment to the liver following systemic DNA administration. Collectively, these findings underscore the importance of early RBC-mediated DNA delivery in driving the host inflammatory response. A failure in this process, as observed in the absence of RBC-TLR9, resulted in attenuated cytokine responses and inflammatory cell recruitment, impairing microbial control.

We observed consistent findings in our clinical cohort where red-cell-associated microbial DNA community richness correlated with IL-6, suggesting that RBC-mediated DNA delivery is a driver of the IL-6 response at the tissue level. In addition, we have previously demonstrated that critically ill patients with sepsis demonstrate elevated surface RBC-TLR9 and diversity in surface RBC-TLR9 expression.(1) These findings lead us to speculate that RBCs contribute to the host inflammatory response and IL-6 signaling and may contribute to host diversity through intrinsically distinct DNA binding capabilities. However, further studies of RBC heterogeneity and surface TLR9 expression in more extensive cohorts will be needed to validate this hypothesis.

Our data show that RBCs can avidly bind microbial DNA from multiple pathogenic organisms *in vitro*. Furthermore, RBCs acquire microbial DNA in a murine model of polymicrobial sepsis and a cohort of critically ill septic patients. We have previously reported that RBCs sequester mitochondrial DNA during non-COVID and COVID sepsis and that RBC-bound mtDNA is associated with anemia.(1, 14) Sequestration of mtDNA was not unexpected as numerous studies have demonstrated the presence of elevated cell-free mtDNA in the plasma of critically ill patients.(15, 16) One study, however, has linked the presence of bacterial DNA in the circulation with outcomes in critically ill patients with COVID-19.(17) The detection of bacterial DNA bound to RBCs in patients with culture-negative sepsis was an unexpected finding but consistent with the literature that demonstrates the presence of cf-bacterial DNA in the circulation of patients with culture-negative sepsis.(18-20) Consistent with these findings, others have found bacterial DNA present in remote organs, including the brain and lung, during sepsis, suggesting that gut microbial translocation contributes to organ injury during sepsis. (21-23)

Although we observed increased 16S DNA on RBCs from septic patients when compared with healthy donors, the detection of microbial DNA present on RBCs in the healthy donors suggests that another homeostatic role of RBCs may be to scavenge microbial DNA from the gut or other tissues. Given, the ample evidence for microbial translocation into the circulation during routine tasks such as tooth brushing, exercise, and simply aging (24-27), it is plausible that RBCs may serve to sequester microbial DNA under basal conditions. Currently, the origin of RBC-bound microbial DNA remains enigmatic. Given that RBCs circulate through all tissues, we hypothesize several potential sources of RBC-bound microbial DNA, including direct acquisition from infected tissue sites, uptake of microbial DNA from the circulation during infection, and intermittent transfer of bacteria through common sources of transient bacteremia. Our studies, comparing bacterial density, diversity, and community composition in patients with both sepsis and culture-negative sepsis, revealed no significant differences. This finding challenges the assumption that bacteremia is the primary source of RBC-bound microbial DNA. In our cohort, probable pathogens constitute only a small portion of the RBC-enriched taxa. This leads us to surmise that the association of bacterial DNA with RBCs may be part of a regulated homeostatic mechanism, wherein the host adjusts its systemic immune response based on the nature of bacteria acquired from these diverse sources. Although speculative at this time, such a mechanism, if confirmed, could redefine our understanding of the interplay between microbial exposure during homeostasis and systemic immune modulation. It is thus not surprising that healthy donors and septic patients exhibited a large amount of nucleic acid on their RBCs. The latter likely reflects the ability of RBCs to sequester DNA from the immediate environment, further supporting the hypothesis that RBCs function to maintain homeostasis by continually scavenging cf-DNA(14). During infection, however, we have recently found that excess CpG-binding leads to innate immune activation and inflammation, and others have validated these findings, demonstrating immunogenicity of CpG-loaded RBCs using *in vivo* tumor models(1, 28). Our current observations suggest that during sepsis, in the presence of excess bacterial DNA, RBCs deliver DNA to remote organs driving inflammation.

RBC-based diagnostics may provide insight into inflammation and injury at the tissue level that is not obtainable from plasma. There is potential that this methodology might lead to novel detection strategies for pathogens using small volume samples as we detected a high abundance of microbial DNA from just $10^7$ RBCs (~ 2uL of RBCs). Identifying an easily accessible, low-volume, high-mass template for molecular diagnostics represents a fundamental breakthrough in pathogen diagnostics. However, as it currently stands, the 16S rRNA bacterial gene sequencing is not an optimal platform to interpret the microbial signal present on RBCs and translate it into actionable bedside information. This is likely for several reasons. One is that despite a high bacterial signal on the RBCs, finding pathogenic DNA appears to be a needle in the haystack problem. The amount of DNA might be overwhelmed by the other more predominant bacterial nucleic acids as described above. Secondly, there is no guarantee that if pathogen DNA is sequestered on the RBC surface it will be the 16S rRNA gene fragment. Additionally, due to the long lifespan of RBCs, it is plausible that microbial DNA associated with the RBCs detected by sequencing reflects microbial DNA present over the lifespan of the RBC (~120 days). As evidenced by our 16S rRNA gene analysis of human RBCs, this low-biomass specimen is also vulnerable to procedural and sequencing contamination. Hence, in the context of diagnostics, the application of RBC-based 16S sequencing is currently considered to have limited effectiveness. Future research focusing on targeted qPCR for detecting RBC-bound pathogens may be more beneficial.

While the quantity of 16S bound to RBCs did not associate with inflammatory cytokines in patients, *community composition* and *richness* of RBC-associated microbial DNA did associate with IL-6. Further *in vitro* mechanistic studies will be needed to interrogate the hypothesis that

RBC-bound microbial DNA composition differentially regulates host inflammatory responses including phagocyte cell death. Our clinical observations of RBC-bound DNA associated with inflammation are validated in the pre-clinical animal model, where we demonstrate that loss of RBC-TLR9 attenuates end-organ IL-6 and IL-1B production in severely ill animals.

In this study, we uncover a new non-gas exchanging function of RBCs as couriers of microbial DNA through TLR9. Our findings show that RBC mediated DNA transport elicits specific host responses in a preclinical sepsis model and RBC-bound microbial DNA associated with systemic inflammation in sepsis patients, offering new insights into previously unexplored mechanisms contributing to the heterogenous host response in sepsis. Additionally, RBCs emerge as an unexpected reservoir for microbial DNA that may be exploited in the future to develop molecular diagnostics for infectious diseases. Collectively, we identify a critical role for circulating RBC-TLR9 in modulating the host inflammatory responses during sepsis, highlighting their dual function as both reservoirs and couriers of microbial DNA.

### METHODS

#### Sex as a biological variable

This study examined male and females in the pre-clinical sepsis model, with similar findings reported for both sexes.

#### Sepsis Cohort

RBCs from the day of ICU admission were obtained from human subjects enrolled in the MESSI cohort study at the University of Pennsylvania (IRB #808542).(1) Patients were eligible if they presented to the medical ICU with strongly suspected or confirmed infection and new or worsening organ dysfunction per historic Sepsis-2 "severe sepsis" criteria because enrollment preceded the publication of Sepsis-3.(29) Exclusion criteria included the primary reason for ICU admission being unrelated to infection, admission from a long-term acute-care hospital that might select for sepsis survivors or desire for exclusively palliative measures on ICU admission. Human subjects or their proxies provided informed consent.

**Experimental animals**

C57BL/6 mice were purchased from the Charles River Laboratories Inc. TLR9 KO mice were produced by S. Akira and provided by E. Behrens (Children's Hospital of Philadelphia). Mice lacking erythrocyte TLR9 ($Ery^{tlr9-/-}$) were generated by crossing ErGFPcre mice (a gift from U. Klingmüller, German Cancer Research Center) and TLR9 KO Conditional mice (obtained from the European Mutant Mouse Archive). Genotype was confirmed through PCR amplification with the primers listed in Supplemental Table 1. All experimental procedures were performed on 8- to 12-week-old mice. Littermate controls lacking Cre expression were used in all experiments where $Ery^{\mathrm{tlr9-/-}}$ mice were used.

**Reagents**

Bacterial genomic DNA was obtained from ATCC (25923D-5 for *S. aureus* subsp. *aureus* strain Seattle 1945 and 47085D-5 for *P. aeruginosa* strain PAO1-LAC, 700721D-5 for *K. pneumoniae* subsp. *pneumoniae* strain MGH78578). *Legionella pneumophila* and *S. pneumoniae* were kind gifts from Dr. Sunny Shin and Dr. Hao Shen, respectively (University of Pennsylvania). The corresponding genomic DNA was extracted using a DNeasy kit (Qiagen). IDT synthesized nucleotides and ODN1826 (CpG).

#### Macrophage isolation

WT mice were injected with 3mL Brewer's thioglycollate media intraperitoneally to obtain thioglycollate-elicited macrophages. After four days, mice were sacrificed via $CO_2$, and peritoneal lavage was aseptically collected by injecting and aspirating 5mL RPMI-1640 media in the peritoneum twice. The lavage was strained through a 70µm cell strainer, and the cells were adhered to a tissue culture-treated dish for 1hr at 37ºC in D10 media (DMEM supplemented with 10% FBS, 1% Pen/Strep, and 1% L-glutamine). Non-adherent cells were removed by washing three times with PBS (with calcium/magnesium). Adherent macrophages were lifted with a cell scraper and seeded onto 24-well plates at 250,000 cells per well overnight at 37ºC in D10 media. Macrophages were washed with PBS once before incubation with RBCs. In some experiments, macrophages were seeded onto sterile 12mm coverslips in 24-well plates.

#### *In vitro* DNA delivery by RBCs

Freshly isolated, leukoreduced RBCs were incubated with DNA at the ratio of 10ng bacterial DNA to $10^7$ RBCs in 200µL DMEM for 4hr at 37ºC on a nutator. 25µg/mL ODN1826 was used as a control. All binding reactions were carried out in DNA LoBind tubes (Eppendorf). Subsequently, the DNA-RBC mixture was added to macrophages and incubated at 37ºC for 4hr. The supernatant containing RBCs were harvested and frozen at -80ºC for ELISA. To evaluate hemolysis, 60µL of the supernatant was centrifuged at 800g for 5min, and 50µL of the clarified supernatant was used to quantify cell-free hemoglobin using QuantiChrome hemoglobin assay according to the manufacturer's instructions. The remaining cells from the clarified supernatant were resuspended in PBS and cytospin onto SuperFrost Plus slides and stained with DiffQuik according to the manufacturer's instructions. To evaluate erythrophagocytosis, macrophages on coverslips were washed in PBS and fixed in 100% methanol. Coverslips were mounted onto glass slides and stained with DiffQuik. For immunofluorescence, methanol-fixed cells were rehydrated in PBS and stained with 1μg/mL Hoechst 33342 (Invitrogen) and 66μM AlexaFluor488-labelled phalloidin (Invitrogen) in PBS supplemented with 1% normal goat serum for 20min at room temperature. Stained cells were washed with PBS, mounted onto Fluoromount-G and imaged with a Nikon 2A microscope.

**DNA binding to human RBCs**

RBCs were isolated by leukoreduction as previously described.[3] RBCs were incubated with 1 or 10ng of bacterial DNA (bDNA) in 200μL PBS in DNA lo-bind tube (Eppendorf) on a nutator at 37ºC for 2hr. The RBCs were then isolated using sucrose gradient centrifugation (30% sucrose cushion, 13,000g for 5 min). The isolated red cell pellets were frozen at -80ºC until DNA extraction with a DNeasy blood kit (Qiagen). After DNA extraction, DNA was eluted in 152μL buffer AE. RBC-associated DNA was quantified with qPCR using either QuantStudio 6 or 7 (Applied Biosystems) using primers and probes listed in Supplemental Table 1. For *S. aureus* or *K. pneumoniae*, 16S multiplex primers were used in conjunction with the corresponding species-specific probe.(30)

**In vivo DNA delivery by RBCs**

Leukoreduced murine RBCs from WT or TLR9 KO mice were washed with PBS and concentrated to a hematocrit of 40%. 160μL of the RBCs were mixed with 50μg ODN1826 at 40μL and transfused into recipient mice intravenously. After 6hr, blood was harvested via cardiac puncture, and plasma cytokines were quantified by ELISA. Livers were fixed in formalin and processed for histology as above.

**Cecal slurry-induced sepsis**

Ery$^{tlr9-/-}$ mice and their littermates that lack Cre expression (controls) were used. Cecal slurry was generated as previously described and injected at 2mg/kg intraperitoneally.(1) Body temperature was monitored using an infrared thermometer. At the indicated time, mice were euthanized with 80 mg/kg ketamine and 10 mg/kg xylazine. Blood was collected via cardiac puncture with heparinized syringes. Tissues were excised and stored in TRIzol (Ambion), 10% buffered formalin acetate or PBS.

Quantification of tissue bacterial load was performed on the same day as necropsy. Tissues were weighed prior to storing in PBS and then homogenized and serially diluted in PBS. 10µL of each dilution was plated onto brain-heart infusion agar in triplicates and incubated at 37ºC for 24hr, and bacterial colonies were counted. Bacterial dissemination was defined as > $10^5$ CFU/tissue. Isolation of RBCs for bound bacteria quantification were purified as previously described using TER119 beads. To generate RBC smears, 5µL of packed blood was spread onto SuperFrost Plus microscope slide. The slides were then air-dried and stained using DiffQuik.

**Histology scoring criteria**

For histology, lungs were vacuum expanded overnight in formalin. Spleens and liver were fixed overnight in formalin. Tissues were then processed for paraffin sectioning and scoring by the Comparative Pathology Core of the University of Pennsylvania School of Veterinary Medicine.

Histopathological examination was performed blinded. 20 fields of 400X tissue sections were evaluated for histology criteria listed in Supplemental Table 1. As no validated sepsis scoring system exists for the liver and spleen, all histological findings were scored in a semiquantitative manner based on severity per International Harmonization of Nomenclature and Diagnostic Criteria (INHAND) recommendations. Criteria considered common findings in sepsis were then weighted higher than less specific lesions and common background changes.

**RBC smear scoring**

The blood smear score was determined by two blinded reviewers using an Olympus BX41 microscope at 40X magnification. Five random fields of view were assessed for each mouse. Echinocytes, bite cells, rouleaux, and teardrop cells were considered 'abnormal' RBCs. If over 50% of the field-of-view contained abnormal cells, then that field-of-view was given a score of one. If less than 50% of the field-of-view contained abnormal cells, then that field-of-view was given a score of zero. Once five random fields-of-view were scored for a given mouse, then a total score from zero to five was determined by summing the individual field-of-view scores for the mouse. A total score of zero indicates a low abnormality, while a total score of five indicates a very high abnormality.

**qRT-PCR**

RNA was extracted from tissues in TRIzol (Ambion) or from RBCs using a RNeasy Plus kit (Qiagen) following the manufacturer's protocol. DNA on RBC samples was extracted with a DNeasy blood and tissue kit (Qiagen). RNA was reverse transcribed into cDNA using a High-Capacity cDNA kit (Applied Biosystems). Gene expression was quantified with PowerUp SYBR

Green Master Mix or TaqMan Fast Universal PCR Master Mix (Applied Biosystems) with primers or probes listed in Supplemental Table 3. For quantification of 16S rDNA on murine RBCs, a 1:5 dilution of DNA was used for qPCR.

**Cytokine quantification**

Cytokine expression in mouse plasma was quantified with a custom U-plex cytokine panel (Meso Scale Discovery) of the following analytes: IL-6, TNFα, IL-1β, IL-10, IL-12p70, IL-27p28, and IFNγ or standard ELISA according to manufacturer's protocol (DuoSet, R&D Systems).

**DNA extraction from cecal slurry**

400mg of cecal slurry stocks were thawed and centrifuged at 5000g to pellet bacteria. DNA were extracted from cecal slurry stocks using QIAmp PowerFecal Pro DNA kit (Qiagen) following the manufacturer's procedure. The bacteria pellet was resuspended in 600μL buffer CD1 and the final elution volume of DNA was 50μL in buffer C6.

**Isolation of F4/80$^{+}$ red pulp macrophages**

F4/80+ cells were magnetically isolated with F4/80-bead (Miltenyi Biotec) following manufacturer's protocol.

**Bioinformatics**

We performed unsupervised learning to identify clusters within the data. We applied agglomerative (hierarchical) clustering to the Z-normalized cytokine data with using a complete linkage to produce heatmaps, and dimensional reduction with uniform manifold approximation and projection (UMAP). For this latter approach, we used the Python package *umap-learn* to calculate the projection using the nine cytokines as input features. To visualize how the underlying features contributed to the visualized clusters we fit a kernel density estimator to the UMAP coordinates weighted by the log of the features Z-scores to produce contour maps. Graphics were generated using a combination of the Seaborn and Matplotlib packages.

**Isolation of Peripheral Blood Mononuclear Cells and Erythrophagocytosis Assays**

Whole blood was obtained from healthy volunteers and was diluted 1:1 in sterile PBS. Diluted blood was layered on top of Ficoll and spun at 2000 G for 20 minutes and PBMCs were collected. The remaining blood was stored in citrate-phosphate-dextrose (CPD, Sigma). $4x10^5$ monocytes were stimulated in a 6-well plate with 2µM 2006 CpG-ODN overnight in D10 media. CPD stored blood was centrifuged at 3000 G and packed RBCs were leukoreduced (Acrodisc, Pall Laboratories). RBCs were manually enumerated and $1x10^6$ were incubated in 50µL PBS or 2006 CpG-ODN DNA for 2 hours at 37°C. RBCs were washed in PBS and resuspended in D10 media prior to incubation with plated PBMC derived monocytes for 15 minutes at 37°C (10:1 RBC to monocyte ratio). Cells were aspirated and RBCs were lysed (ACK lysis buffer). Cells were washed x2 prior to blocking with Fc Block for 30 mins. Cells were fixed with BD Cytofix/Cytoperm for 20 minutes, washed 2x in FACS buffer, and intracellularly labeled with either PE anti-human Glycophorin A (CD235a, Biolegend) or PE mouse IgG2a isotype for 1 hour. PBMCs were washed twice in FACS buffer. FACS acquisition and analysis was performed on an LSR Fortessa.

**16S sequencing and analysis**

The 16S rRNA amplicon sequences were initially analyzed using QIIME2 (qiime2.org, version 2021.2). DADA2 was used for quality control, denoising, and amplicon sequence variant (ASV) creation.(31) ASV with less than 10 hits across samples were filtered out. A naïve Bayesian classifier was used to assign taxonomy against the Greengenes (version 13.8) database. We performed microbial ecology analysis using the *vegan* package 2.6-1 and in *R* 4.2.2.(32-34) For relative abundance and ordination analysis, samples were normalized to the percent of total reads and we restricted analysis to ASVs that were present at greater than 1% of the sample population. All ASVs were included in diversity analysis. Direct community similarity comparisons were performed using the Bray-Curtis similarity index. We performed ordinations using Principal Component Analysis on Hellinger-transformed normalized OTU tables generated using Euclidean distances(35). We determined significance of differences in community composition using PERMANOVA (*adonis*) with 10,000 permutations using Euclidean distances. We compared means via Student's T test and ANOVA with Holm-Sidak's multiple comparisons test as appropriate.

**Statistics**

GraphPad Prism was used to perform all statistical analysis with the exception of 16S sequencing analysis and Bioinformatics analysis described above. T-test or Mann-Whitney U-test was used to compare between 2 groups depending on normality. One-way ANOVA or Kruskal-Wallis was used to compare more than two groups depending on normality, followed by indicated post hoc tests.

**Study Approval**

All studies involving animals were approved by the University of Pennsylvania IACUC. For studies involving human subjects, all studies were approved by the University of Pennsylvania IRB. Patients or their proxies provided written informed consent.

**Data Availability:** All data are available in the main text or the supplementary materials. Raw sequence data is uploaded to the NCBI sequence read archive (SRA).

**Author contributions:** Experiments were conceived and designed by LKML and NSM. Experiments were performed by LKML, KAE, ML, EAO, LS, and JSJ. Data were analyzed by LKML, JSJ, CVC, JM, NK, NY, NJM, PR, RPD and NSM. NSM and RPD wrote the paper.

**Acknowledgments:**

**Funding:**

NIH R01 HL126788 (NSM)

NIH R21 AI166813 (NSM)

Penn Synergy Program, Penn Center for Precision Medicine (NSM).

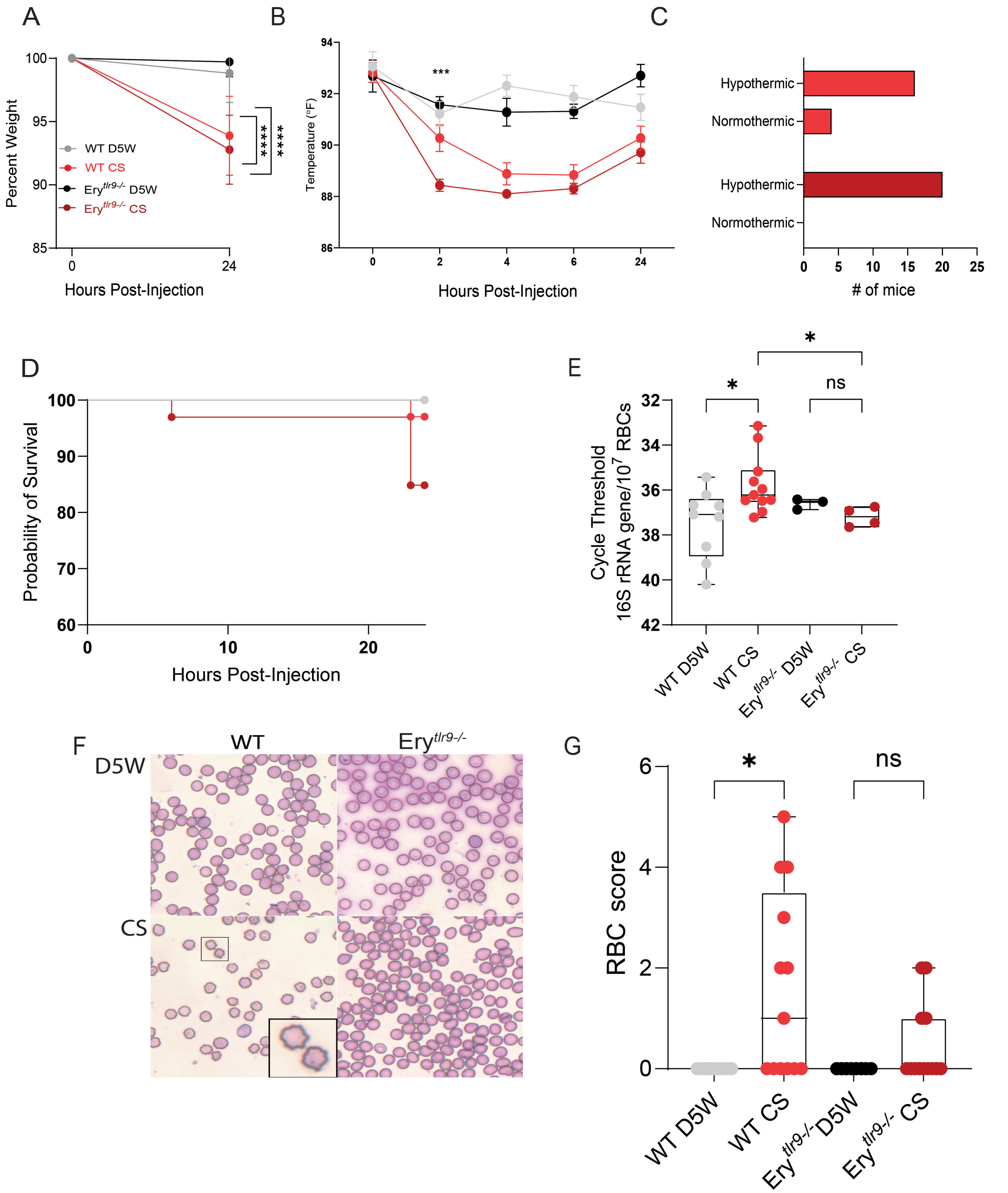

**Fig. 1. RBCs from WT but not Ery$^{tlr9-/-}$ mice acquire microbial DNA and demonstrate morphologic changes during sepsis.** Ery$^{tlr9-/-}$ or WT littermates were injected with CS or D5W and monitored for 24 hours. (**A**) Weight change and differences between groups were analyzed by ANOVA with Sidak's multiple comparisons, ****P<0.0001 for WT D5W v WT CS and Ery$^{tlr9-/-}$ D5W v Ery$^{tlr9-/-}$ CS, WT CS v Ery$^{tlr9-/-}$ CS= ns, n=20-30 mice per group. (**B**) Temperature profiles of injected mice, 88°F was the lower limit of detection, ANOVA with Sidak's multiple comparison analysis, ***P=0.002 between CS-injected groups at 2 hours, P=0.005 between Ery$^{tlr9-/-}$ D5W and Ery$^{tlr9-/-}$ CS, n=9-20 mice per group. (**C**) Hypothermic state of the mice by strain at 24 hours, P=0.035 by chi-square test. (**D**) Kaplan Meier survival with log-rank comparison, P=0.031 between all groups, P=0.08 WT CS v Ery$^{tlr9-/-}$ CS. (**E**) RBC-associated 16S rRNA gene on murine RBCs was quantified 6 hours following CS-induced sepsis. One-way Kruskal-Wallis test with Dunn's multiple comparisons, n=3-11, *P =0.042 WT D5W v WT CS, *P=0.044 WT CS v Ery$^{tlr9-/-}$ CS. (**F**) RBCs from WT or Ery$^{tlr9-/-}$ mice 24 hours following D5W or CS injection, insert shows echinocytic RBCs observed in the WT CS injected mice(**G**) RBC score (number of echinocytes and altered RBCs/hpf), P= 0.013, WT D5W v WT CS, n=8-14 from 3 independent studies.

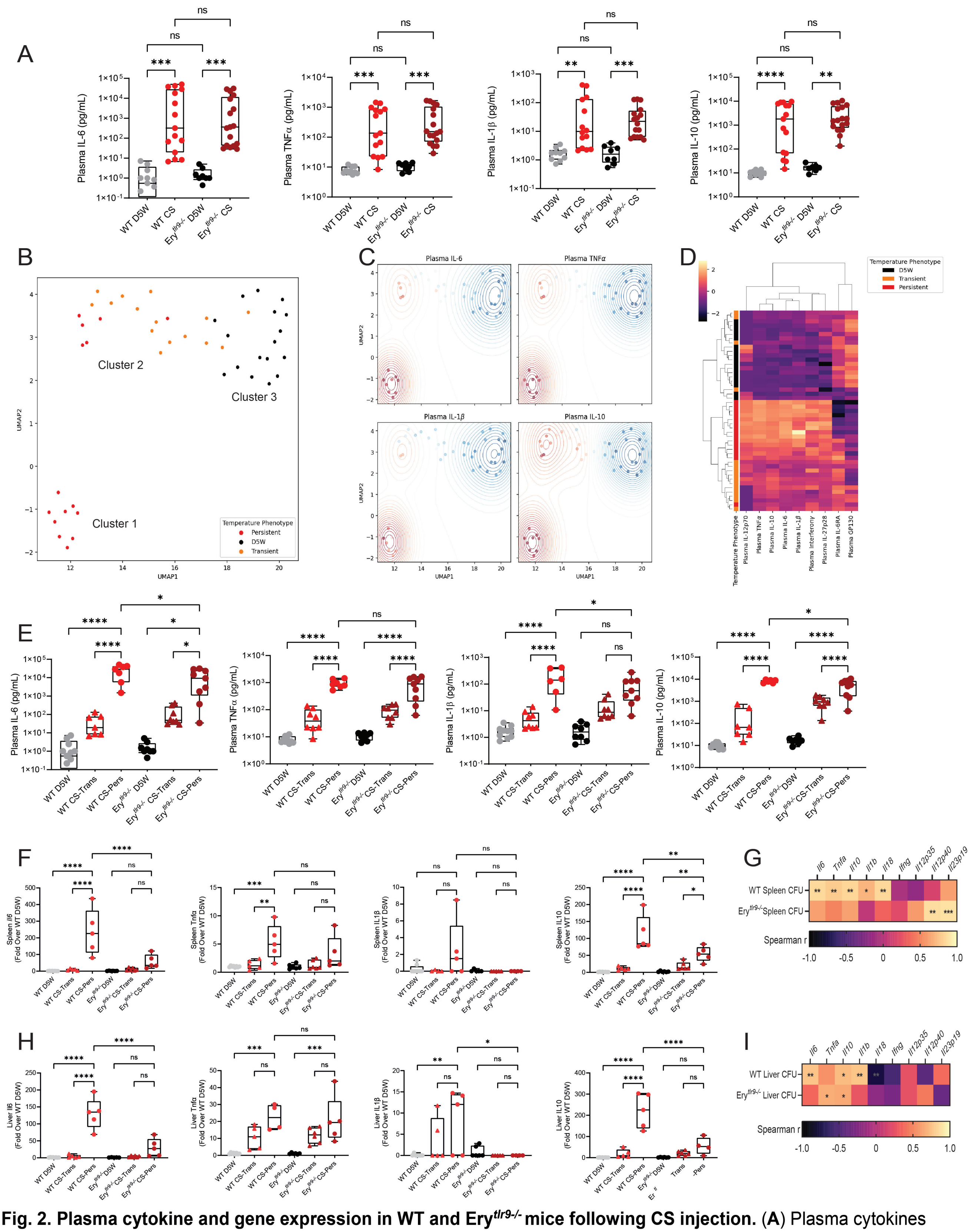

**Fig. 2. Plasma cytokine and gene expression in WT and Ery$^{tlr9-/-}$ mice following CS injection.** (**A**) Plasma cytokines 24 hours following CS or D5W injection, n=8-17 per group from 4 independent studies. (**B**) Uniform manifold approximation and projection derived from the nine measured plasma cytokine concentrations colored by whether they developed persistent or transient hypothermia or were controls (D5W). (**C**) Weighted kernel density estimation illustrating relative concentration (red higher, blue lower) of the cytokines in each cluster. (**D**) Heatmap of cytokines structured with agglomerative clustering similarly illustrating the correlation between cytokine concentration and hypothermia. (**E**) Plasma cytokines 24 hours after CS or D5W injection, plasma cytokines stratified based on hypothermic state. (**F**) Splenic expression of immune genes stratified based on hypothermic state and (**G**) their correlations to splenic bacterial load. (**H**) Hepatic expression of immune genes stratified based on hypothermic state and (**I**) their correlations to hepatic bacterial load. Differences between groups were analyzed by one-way Kruskal-Wallis test with Dunn's post-hoc in (**A**) and one-way ANOVA with Holm-Sidak test in (**E**), (**F**), and (**H**), n=4-8 per temperature stratified group from 4 independent studies in (**F-I**). Association between bacterial load and tissue cytokines,Spearman's correlation. *P <0.05, **P< 0.01, ***P<0.001, ****P<0.0001.

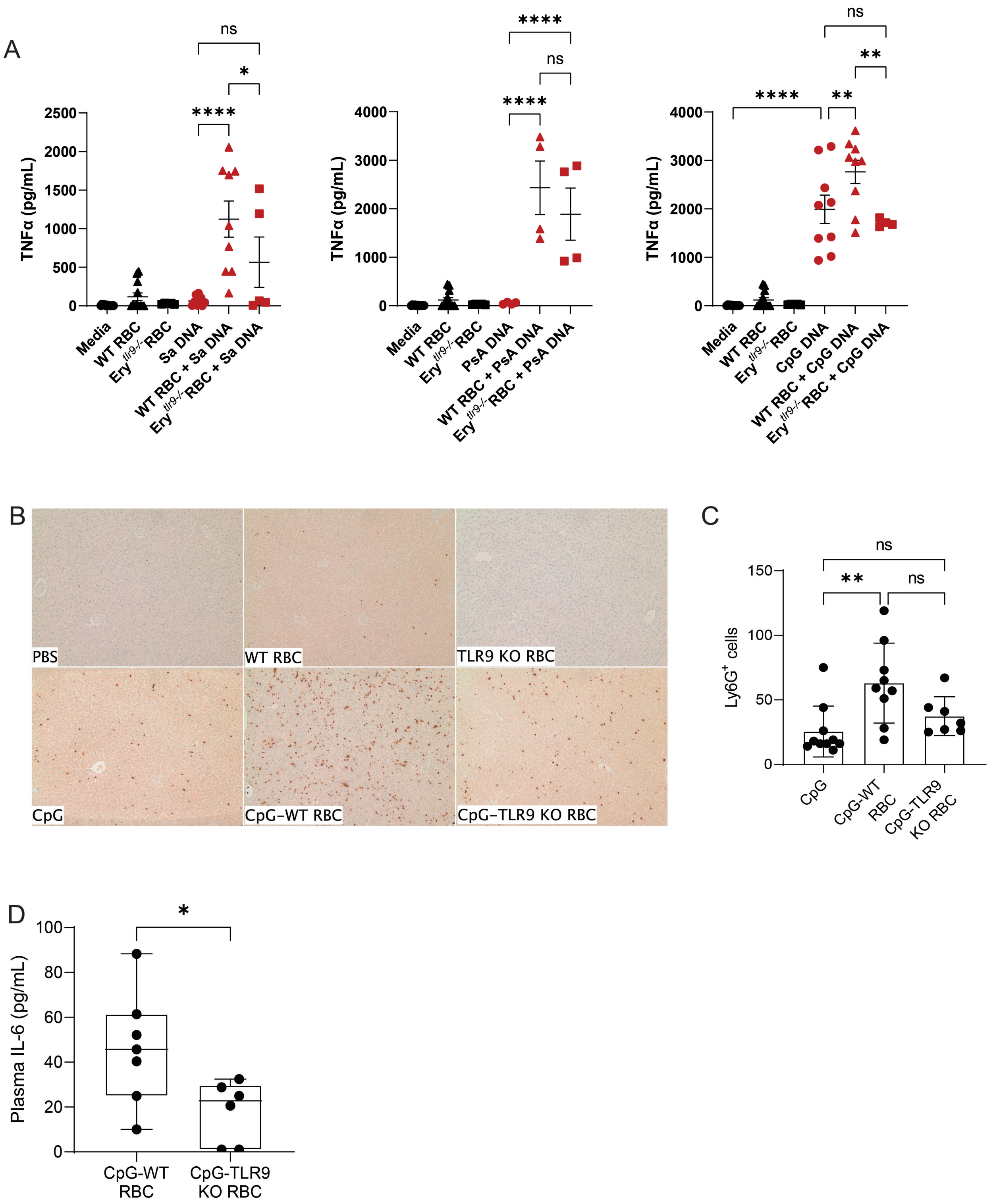

**Fig. 3. Murine RBCs acquire and deliver DNA to remote organs and immune cells** (**A**) TNFα production by peritoneal macrophages four hours following treatment with media, RBCs (WT or Ery$^{tlr9-/-}$), or RBCs pre-incubated with *S. aureus* genomic DNA (Sa DNA), *P. aeruginosa* DNA (PsA), or CpG-ODN 1826. Results from 2-4 independent studies are shown. Differences between groups were analyzed with one-way ANOVA followed by Holm-Sidak post-hoc comparison with *P<0.05, **P<0.01 and ****P<0.0001. (**B**) Ly6G staining of liver sections 6 hours following transfusion of CpG or CpG-treated RBCs and (**C**) quantification of Ly6G$^{+}$ cells. (**D**) Plasma IL-6 6 hours following transfusion of mice with CpG-treated WT or TLR9 KO RBCs. Differences between groups were analyzed with Kruskal-Wallis test and Dunn's posthoc analysis in (**B**) and unpaired t-test in (**D**), n=6-10 from 2 independent experiments, *P<0.05 and **P<0.01.

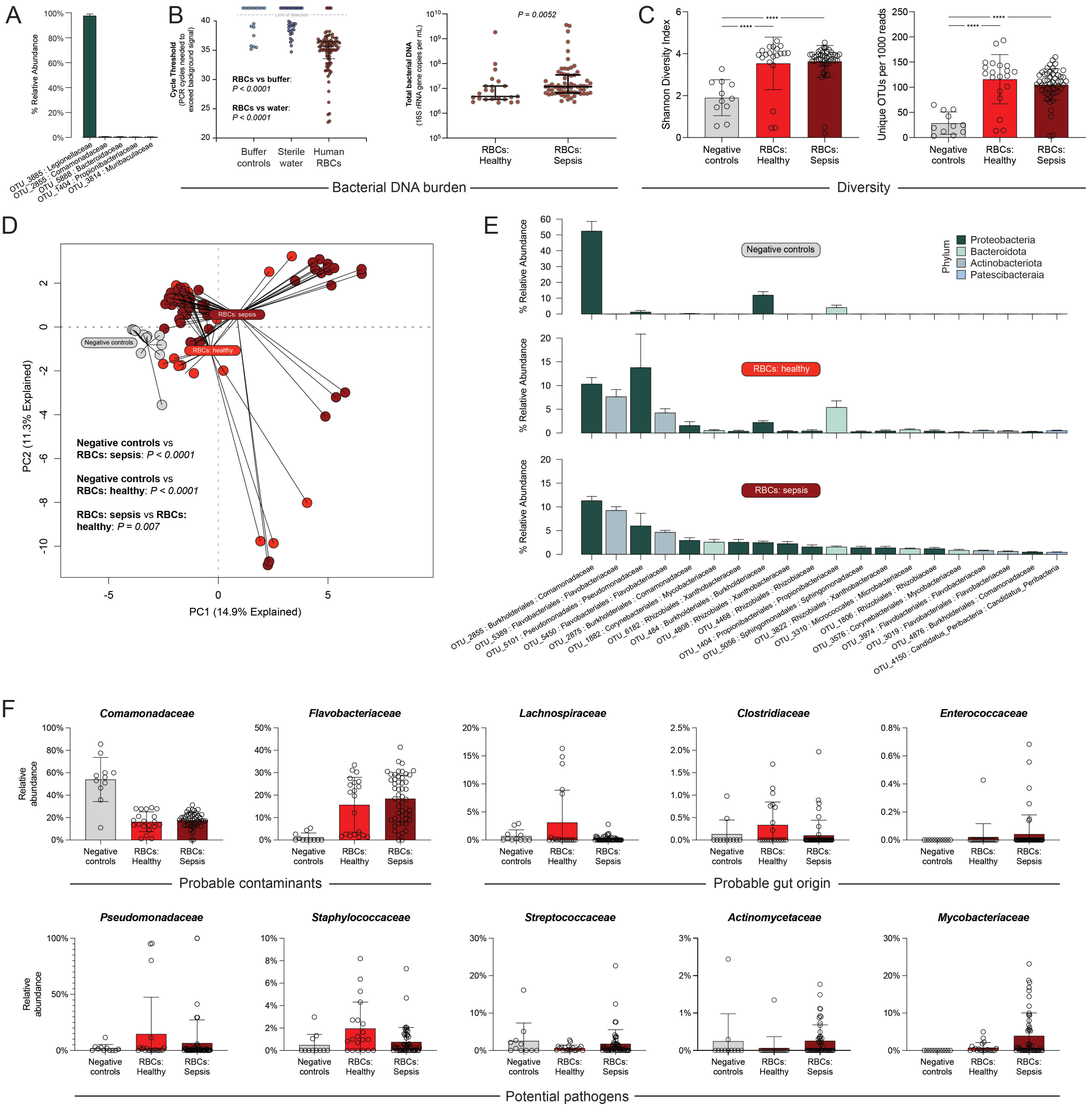

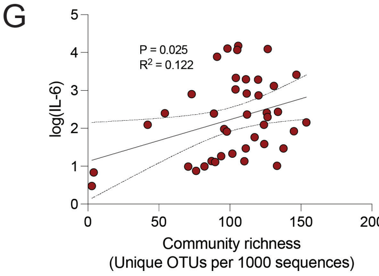

**Fig. 4. Analysis of bacterial DNA associated with red blood cells.** (**A**) We incubated red blood cells with Legionella sp. then performed 16S rRNA gene amplicon sequencing on the red blood cells. RBC-associated DNA was dominated by Legionella-classified amplicons (97.5%). (**B**) We quantified RBC-associated bacterial DNA using qPCR of the 16S rRNA gene. Human RBCs had a greater quantity of bacterial DNA than did negative control specimens, and RBCs from humans with sepsis had more bacterial DNA than healthy volunteers. (**C**) RBC-associated bacterial DNA contained a greater diversity of bacterial taxa than did negative control specimens. (**D**) Bacterial taxa detected in RBCs (both in health and sepsis) were distinct from those of negative control specimens and distinct from each other. (**E**) Rank abundance analysis demonstrated the influence of some contaminant taxa on RBC taxa (e.g., Comamonadaceae) as well as distinct taxa within RBC specimens not detected in negative control specimens. (**F**) Direct comparison of prominent bacterial families across negative controls and RBC from healthy and septic patients. (**G**) Among patients with sepsis, the acute inflammatory cytokine IL-6 was positively correlated with community richness.

**Table 1. Organ dissemination stratified by strain.** Data are shown as number observed (%). Comparisons shown were calculated by chi-square tests. For the blood dissemination, n=18 for WT and 15 for Ery$^{tlr9-/-}$.

| | **WT** | **Ery$^{tlr9-/-}$** | ***P*** |
|---|---|---|---|
| | **(n=19)** | **(n=16)** | |
| Blood | 8 (44%) | 11 (65%) | 0.09 |
| Spleen | 10 (53%) | 14 (88%) | 0.03 |
| Liver | 13 (68%) | 15 (94%) | 0.06 |
| Lung | 13 (68%) | 13 (81%) | 0.39 |

**Table 2. MESSI cohort patient characteristics.**

| | n (%), median [IQR] |
|---|---|
| Age (median, IQR) | 61 [48.5, 68.5] |
| Gender (%female) | 17 (33%) |
| Admission source: | |
| ED | 28 (55%) |
| Transfer | 19 (37%) |
| OSH transfer | 4 (8%) |
| Comorbidities | |
| ARDS | 15 (29%) |
| Lung source of sepsis | 29 (57%) |
| 30 day mortality | 19 (37%) |
| 90 day mortality | 27 (53%) |
| Blood Culture Positive | 17 (33%) |

## Supplementary Files

This is a list of supplementary files associated with this preprint. Click to download.

- SupplementalInformation.pdf
